# Current Evidence Linking Polyunsaturated Fatty Acids with Cancer Risk and Progression

**DOI:** 10.3389/fonc.2013.00224

**Published:** 2013-09-04

**Authors:** Maria Azrad, Chelsea Turgeon, Wendy Demark-Wahnefried

**Affiliations:** ^1^Department of Nutrition Sciences, University of Alabama at Birmingham, Birmingham, AL, USA; ^2^School of Medicine, University of Alabama at Birmingham, Birmingham, AL, USA; ^3^Comprehensive Cancer Center, University of Alabama at Birmingham, Birmingham, AL, USA

**Keywords:** polyunsaturated fatty acids, cancer, pre-clinical testing, epidemiologic studies, clinical trials as topic, clinical trials, phase II as topic, review of literature

## Abstract

There is increasing evidence that polyunsaturated fatty acids (PUFAs) play a role in cancer risk and progression. The *n*-3 family of PUFAs includes alpha-linolenic acid (ALA), eicosapentaenoic acid (EPA), and docosahexaenoic acid (DHA) while the *n*-6 family includes linolenic acid (LA) and arachidonic acid (AA). EPA and DHA are precursors for anti-inflammatory lipid mediators while AA is a precursor for pro-inflammatory lipid mediators. Collectively, PUFAs play crucial roles in maintaining cellular homeostasis, and perturbations in dietary intake or PUFA metabolism could result in cellular dysfunction and contribute to cancer risk and progression. Epidemiologic studies provide an inconsistent picture of the associations between dietary PUFAs and cancer. This discrepancy may reflect the difficulties in collecting accurate dietary data; however, it also may reflect genetic variation in PUFA metabolism which has been shown to modify physiological levels of PUFAs and cancer risk. Also, host-specific mutations as a result of cellular transformation could modify metabolism of PUFAs in the target-tissue. Clinical trials have shown that supplementation with PUFAs or foods high in PUFAs can affect markers of inflammation, immune function, tumor biology, and prognosis. Pre-clinical investigations have begun to elucidate how PUFAs may mediate cell proliferation, apoptosis and angiogenesis, and the signaling pathways involved in these processes. The purpose of this review is to summarize the current evidence linking PUFAs and their metabolites with cancer and the molecular mechanisms that underlie this association. Identifying the molecular mechanism(s) through which PUFAs affect cancer risk and progression will provide an opportunity to pursue focused dietary interventions that could translate into the development of personalized diets for cancer control.

## Introduction

Despite major advances in prevention, screening, and treatment, cancer remains a major public health burden. In the US, it is estimated that over 800,000 cases of cancer will be diagnosed and more than 270,000 people will die from cancer this year (1). Diet is recognized as an important environmental factor contributing to risk and mortality for several types of cancers (2). Specifically, a Western diet which is characterized by high intakes of *n*-6 polyunsaturated fatty acids (PUFAs), and lower intakes of *n*-3 PUFAs, has been suggested to play a role in carcinogenesis and cancer outcomes (3, 4). PUFAs are substantial components of the diet, comprising approximately 7–10% of daily energy intake in US adults (5, 6). Adequate intake of PUFAs is essential as they are biologically active molecules that serve as structural components of cellular membranes and play key roles in metabolism, inflammation, cell signaling, and regulation of gene expression (7).

Given that PUFAs are prominent dietary constituents and indispensable cellular components, a large body of research has been conducted in humans, animals, and in *in vitro* experiments in order to elucidate the link between PUFAs and cancer. Pre-clinical mechanistic studies using animal models and cancer cell lines have begun to elucidate molecular targets of PUFAs, yet these findings have not necessarily translated in human studies. Pre-clinical models use well-characterized cell lines for *in vitro* studies and *in vivo* studies in animal models also show little variation given the use of inbred strains exposed to well-characterized carcinogens or the use of well-characterized xenografts. However, neoplasia in humans is much different. In humans, cancer results from the interaction between diverse genetic backgrounds and a multitude of diverse exposures. Unsurprisingly, the tumors in humans tend to exhibit high genetic/genomic heterogeneity, and recent studies assessing PUFA intake and cancer risk and progression in prospective cohort studies have produced inconsistent results (8). Studies in cardiovascular disease have observed that genetic variation in the form of single nucleotide polymorphisms (SNPs) greatly influences metabolism of PUFAs (9). Findings from these studies could potentially explain some of the inconsistencies observed between dietary intake of PUFAs and cancer in epidemiological studies. Next-generation sequencing (NGS) and better understanding of the cancer genome may inform whether heterogeneity in the host or in the tumor influences PUFA metabolism within individuals, the microenvironment, or the target tissue. If aberrant PUFA metabolism as a result of genetic variation plays a role in cancer risk or genomic changes alter PUFA metabolism in the target tissue and promote cancer progression, it is plausible that personalized-diets could be a therapeutic approach to provide specified intakes of PUFAs based on an individual’s metabolic capability and physiological needs.

## Overview

As reported by Blasbalg et al., radical changes occurred in the American diet during the twentieth century (10). From 1909 to 1999, there was a steady decline in the consumption of animal fat, a major source of saturated fat, as the intakes of butter and lard decreased. This paralleled the significant rise in intake of PUFAs as the consumption of soybean and other vegetable oils increased. It is estimated that from 1986 to 1999, the intake of linolenic acid (LA) and alpha-linolenic acid (ALA) increased >1000- and >100-fold, respectively, as a result of vegetable cooking oils becoming commercially available (10). Although significant changes in intake of essential PUFAs is well documented, it should be noted that intakes of arachidonic acid (AA), found in animal products, and eicosapentaenoic acid (EPA), docosopentaenoic acid (DPA), and docosahexaenoic acid (DHA), found in fatty fish, remained steady (10).

**Figure 1:**
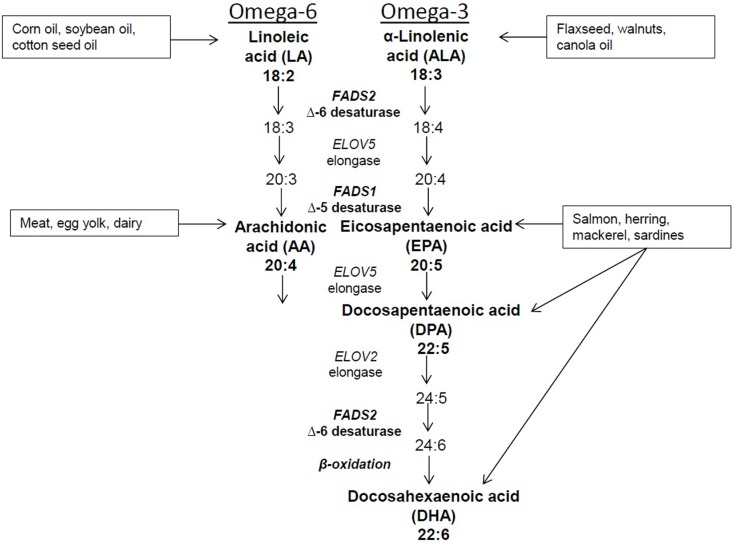
**Dietary sources and metabolic pathway of PUFAs**.

Dietary intake and changes in sources of fatty acids can affect the complex metabolism of PUFAs. In addition, desaturase enzymes encoded by *FADS2* and *FADS1*, as well as elongase enzymes encoded by *ELOV5* and *ELOV2* genes also affect metabolism (Figure 1). The *FADS* genes localize to chromosome 11 at 11q12–q13.1 a region of the genome that is highly polymorphic. The most recent data indicate that there are 330 and 942 identified SNPs in *FADS1* and *FADS2*, respectively (http://www.ncbi.nlm.nih.gov/snp/). Ethnic differences in *FADS* SNPs have been reported as evidenced by recent studies in Caucasian, Asian, and African-American populations (11, 12) It is estimated that the prevalence of variant genotypes is lower in Hispanics (3.6%) and non-white Hispanics (3.1%) compared to Asians or Pacific Islanders (19.4%), blacks (24.0%), and other racial or ethnic groups (18.2%) (13). Based on the findings from several studies, SNPs in *FADS 1* and *2* are associated with physiological levels of PUFAs suggesting that genetic variation plays a strong role in PUFA metabolism and possibly risk for disease.

Because humans cannot synthesize 18-carbon PUFAs, LA, and ALA are considered essential *n*-6 and *n*-3 PUFAs, respectively. LA is the predominant PUFA in the Western diet, and it is converted to the 20-carbon AA. This occurs through removal of two hydrogen atoms and the addition of a double bond between two carbon atoms at the sixth position by the delta-6 desaturase (D6D) enzyme and the addition of a carbon atom at the carboxyl end of the molecule by the elongase enzyme. The metabolism of ALA requires the same enzymes as LA; thus, there is competition between these PUFAs for desaturation and elongation. D6D has a higher affinity for ALA, yet the greater intake of LA seen in the Western diet tends to pull the pathway in favor of *n*-6 PUFA metabolism. However, some dietary ALA is converted via D6D and elongase to yield the 20-carbon EPA, which can be further elongated to the intermediate 22-carbon DPA and ultimately to 22-carbon DHA. As noted earlier, EPA, DPA, and DHA also can be obtained through dietary intake of marine-derived fish, particularly salmon, herring, mackerel, and sardines. Intake of these types of fish is typically low in a Western diet, therefore resulting in a lower intake of longer chain PUFAs and increasing the need for ALA to be converted to EPA and DHA.

In this report, we examine the current results from pre-clinical studies that aimed to elucidate the underlying mechanisms linking PUFAs to cancer. We review the observations from recent prospective cohort studies that investigated associations between dietary intake of PUFAs and cancer risk and progression, and we summarize the recent findings from clinical trials that tested the effects of PUFA supplementation on cancer patients.

## Review of the Literature

### Pre-clinical studies that contribute to our understanding potential mechanisms

Inflammation has been identified as a hallmark of tumorigenesis (14). Studies using *in vitro* techniques and animal models have elucidated the anti- and pro-inflammatory mechanisms through which PUFAs can mediate cancer promotion and progression. Below, we summarize the evidence from studies that have expanded on molecular mechanisms underpinning the relationship between PUFAs and cancer.

Alpha-linolenic acid primarily functions as a precursor molecule for the metabolism of longer chain *n*-3 PUFAs, EPA, and DHA, but it also has been proposed to possess some independent biological functions. The findings from these studies have been inconsistent and the role ALA plays in cancer remains unclear. One study in hepatocellular carcinoma cells showed that ALA induced gene expression of MEK1 and MEKK1, both drivers of cellular proliferation (15, 16). In contrast, other studies have shown that ALA induces apoptosis in estrogen-receptor positive (MCF-7) and negative breast cancer cells (MDA-MV 231) as well as cervical cancer cells, via reduced nitric oxide and increased lipid peroxidation (17). A similar study in breast cancer cells showed that ALA upregulates pro-apoptotic protein Bax, reduces expression of anti-apoptotic Bcl-2, and increases the activation of caspase-3 and proteolytic cleavage of poly(ADP-ribose) polymerase (PARP), all indicators of apoptosis (18).

Similarly, a high ALA diet has been shown to increase apoptosis of hepatoma cells implanted in rats which also correlates with reduced tumor composition of AA and decreased expression of cyclooxygenase-2 (COX-2) (19). Because large amounts of ALA are found in walnuts and flaxseed, these food sources have been examined in several mouse models. To ascertain potential effects on breast cancer, Hardman and Ion employed a nude mouse model implanted with MDA-MB 231 cells and found significantly reduced tumor size in animals fed walnuts compared to controls (20). The same investigators recapitulated the experiment in a transgenic mouse model of breast cancer and found similar results (21). Significant reductions in tumor size were also seen in animals fed walnuts compared to controls in studies using either transgenic animal models of prostate cancer (i.e., the transgenic adenocarcinoma of the mouse prostate (TRAMP) model (22), or xenografts of colon cancer (i.e., HT-29 cells) (23). None of the studies using walnuts found increased apoptosis, but one did observe significant reductions in serum vascular endothelial growth factor (VEGF) and tumor angiogenesis (CD34) (23). It should be noted that walnuts and flaxseed are whole foods that contain other dietary components such as lignan, which also could be associated with reduced tumor proliferation (24) and affect findings.

While *n*-6 PUFAs are collectively thought to be pro-inflammatory, recent studies show that the LA-derived metabolite, 13-S hydroxyoctadecadienoic acid (13-S HODE), produced via 15-lipoxygenase (LOX), is a signaling molecule that has been associated with anti-carcinogenic properties (25). 15-LOX is a proposed tumor suppressor gene and loss of expression is frequently seen in cancer cells (26). Loss of 15-LOX results in reduced levels of intracellular 13-S HODE and higher levels of LA being metabolized to AA which is pro-carcinogenic as detailed below. 13-S HODE mediates anti-carcinogenic activities in lung and colon cancer cells through the activation of peroxisome proliferator-activated receptor (PPAR)-gamma, a nuclear receptor that regulates transcription of several genes (27, 28). Importantly, PPAR-gamma is anti-inflammatory through inhibition of its downstream target NFκB which results in reduced production of pro-inflammatory cytokines, interleukin (IL)-6, and tumor necrosis factor (TNF)-alpha as well as pro-angiogenic VEGF (29). Based on the evidence, LA metabolized by 15-LOX to produce 13-S HODE appears to be anti-inflammatory and may be cancer-protective.

**Figure 2:**
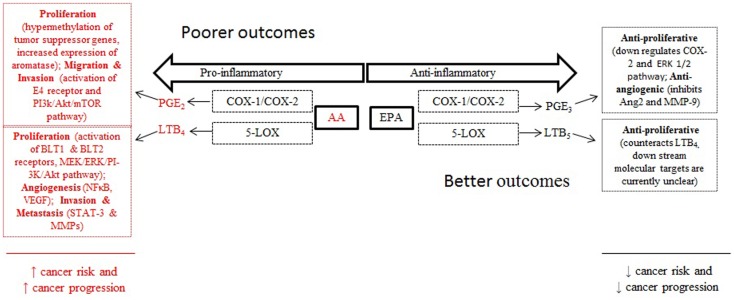
**Arachidonic acid and eicosapentaenoic acid metabolism contribute to cancer risk and progression through pro-and anti-inflammatory lipid metabolites that stimulate cell proliferation, angiogenesis, and migration**.

20-Carbon PUFAs metabolized by the LOX and COX pathways produce leukotrienes, thromboxanes, prostacyclins, and prostaglandins. Collectively, these metabolites have been implicated in several chronic diseases including arthritis, asthma, eczema, and atherosclerosis (30). The metabolites most predominantly linked with cancer are leukotrienes and prostaglandins as they have been shown to play important roles in the progression of cancer through angiogenesis, cell proliferation, metastasis, and apoptosis (31) (Figure 2). The enzyme 5-LOX converts AA to leukotriene B_4_ (LTB_4_). Under normal physiological conditions 5-LOX is not typically expressed but it is upregulated during inflammation and tumorigenesis. As such, LTB_4_ levels have been shown to be higher in human colon and prostate cancer tissues (28). Further, LTB_4_ can act as a growth factor by interacting with the G protein-coupled receptors BLT1 and BLT2. Overexpression of BLT2 has been reported in pancreatic cancer cells where activation of BLT2 by LTB_4_ stimulated pancreatic cellular proliferation through the MEK/ERK and PI-3 kinase/Akt pathways (32). Additionally, LTB_4_ influences the microenvironment by activating NFκB resulting in IL-1, IL-6, and TNF-alpha production and expression of VEGF and angiogenesis (33, 34). Studies in ovarian cancer cells have shown that activation of BLT2 by LTB_4_ stimulates invasion and metastasis through activation of STAT-3 and transcription and synthesis of matrix metalloproteinases (35). Similar findings were observed in prostate cancer cell lines where LTB_4_ activation of BLT2 resulted in NFκB activation and increased expression of the androgen receptor (36). In colon cells, the blockade of BLT1 inhibited cellular proliferation and induced apoptosis though inhibition of the ERK pathway (37).

The predominant AA metabolite produced in the COX pathway is prostaglandin E_2_ (PGE_2_) which has been shown to be cancer promoting in various tissues (38). In normal tissues, COX-1 is constitutively expressed at low levels and COX-2 is undetectable but is inducible during inflammatory response. In cancer cells, COX-2 is frequently overexpressed resulting in the production of high levels of PGE_2_ (31). The non-steroidal anti-inflammatory drugs (NSAIDS) and selective COX-2 inhibitors exert chemoprotective benefit by targeting the COX pathway and reducing the synthesis of PGE_2_ (39). Modulation of dietary PUFAs also have been shown to alter PGE_2_ production in animal models (30, 40). A key mechanism underlying PGE_2_’s capacity to modulate cancer progression is through epigenomic modification. In colorectal cancer cells, PGE_2_ was found to increase the expression of DNA methyl transferase (DNMT)-1 and DNMT3 which resulted in hypermethylation of the promoter regions and reduced RNA and protein expression of tumor suppressor genes (41). This same study reported similar findings in Apc^Min/+^ mice in which PGE_2_ increased DNMT expression, hypermethylation of tumor suppressor genes and accelerated tumor growth (41). PGE_2_ has been linked with breast cancer through its capacity to increase mRNA expression and protein levels of aromatase enzyme which converts androgens to estrogen in breast cancer cells (42). This effect appears to occur through PGE_2_’s stimulation of the receptors E2 and E4 and downstream activation of the cAMP/PKA/CREB pathway. Ultimately, the result is an increase in transcription of the aromatase gene resulting in increased localized estrogen biosynthesis, a key driver of estrogen-receptor positive breast cancer (43). In mouse models, PGE_2_ has been shown to promote angiogenesis and metastatic disease to the lung, whereas blockage of COX-2 results in reduced biosynthesis of PGE_2_ and reduced spread of disease (44, 45). In the prostate cancer cell line, PC3, treatment with TGF-β induced COX-2 expression and PGE_2_ biosynthesis which activated E4 and stimulated the PI3k/Akt/mTOR pathway (46). This resulted in increased migration and invasion of PC3 cells. In addition, PGE2 has been shown to induce VEGF-mediated angiogenesis in PC3 cells through activation of E2 and E4 receptors (47).

Eicosapentaenoic acid, which can be derived from metabolism of ALA or through the diet, is anti-inflammatory on two levels. First, EPA competes with its *n*-6 isomer, AA, for metabolism by COX and LOX enzymes thus reducing the amount of pro-inflammatory PGE_2_ and LTB_4_ synthesized. Second, EPA metabolism by the COX pathway yields PGE_3_, while the LOX pathway yields LTB_5_. In contrast to the actions of PGE_2_, PGE_3_ does not induce cancer cell proliferation and instead down-regulates expression of COX-2 (40). In a mouse model of metastatic colon cancer, animals consuming EPA in comparison to controls had tumors which had lower levels of PGE_2_ and higher levels of PGE_3_, as well as reduced phosphorylated ERK 1/2 expression; moreover, EPA-fed animals also had lower cell proliferation and tumor burden compared to animals on a normal diet (48). PGE_3_ has also been shown to hinder angiogenesis by inhibiting induction of angiopoietin-2 (Ang2), matrix metalloprotease-9 (MMP-9), and endothelial invasion (49). Finally, while fewer studies have focused on LTB_5_ derived from the metabolism of EPA by 5-LOX, it is proposed to counteract the actions of AA-derived LTB_4_ and has been shown to be anti-proliferative in a mouse model for melanoma (50, 51).

Dietary DHA has been shown to significantly reduce tumor size in a dose-response manner in a mouse model of breast cancer (52). This same study also showed that mice consuming DHA and treated with cisplatin had reduced tumor size and enhanced immune response compared to animals receiving cisplatin alone. This was attributed to reduced levels of oxidative stress seen in animals receiving DHA (52). Similar findings have been reported in other studies using DHA and anthracycline and radiotherapy (53, 54).

### Epidemiologic studies that contribute to our understanding of PUFAs and cancer risk (prospective cohort studies)

We identified seven prospective cohort studies that have been published within the past 5 years that investigated the association between PUFAs (either specific fatty acids or mixtures) and risk for cancer or advanced disease. Details of these investigations and major findings are presented in Table 1. Four studies were in US populations including the National Institute of Health–American Association of Retired Persons (NIH-AARP) Diet and Health Study (55, 56), the Multiethnic Cohort Study (57), Cancer Prevention Study-II (CPS-II) (58), and the VITamins And Lifestyle (VITAL) Cohort study (59). One investigation was in a cohort of French women from the E3n study (60) and two studies were in Asian populations including the Shanghai Women’s Health Study (61, 62) and the Japan Public Health Center (JPHC)–Based Prospective Study (63). In all investigations, dietary intake of PUFAs was determined by diet history questionnaire (60) or food frequency questionnaire and was characterized by study-specific PUFA intake according to tertiles (61), quartiles (58), or quintiles (55, 57, 59, 60, 62, 63). Multivariate-adjusted models were used to calculate hazard ratios (HR) or relative risk (RR) and 95% confidence intervals (CIs) to estimate cancer risk associated with PUFA intake. Studies included associations between PUFAs and risk for breast (57, 59–61), colorectal (58, 62, 63), prostate (55), and pancreatic cancer (56). Below, the findings from these investigations are summarized.

**Table 1:** **Prospective cohort studies assessing dietary PUFAs and associations with cancer**.

Cohort	Cancer site	Sample size	Incidence cases	Approximate length follow-up	Findings	Model adjustment
**NIH-AARP** Pelser et al. (55) and Thiébaut et al. (56)]	Pancreas	308,736 men and 216,737 women	865 men and 472 women	6.3	AA, DHA, and total *n*-3 significantly positively associated with risk for pancreatic cancer (HR 1.33 95% CI = 1.12–1.58, *p* = 0.002; HR 1.25 95% CI 1.05–1.49, *p* = 0.009; HR 1.21 95% CI 1.21 95% CI 1.02–1.44, *p* = 0.01 respectively)	Age, energy, smoking, BMI, and diabetes
	Prostate	288,268	23,281	9	ALA significantly positively associated with risk for advanced prostate cancer (HR 1.17 95% CI 1.04–1.31, *p* = 0.01). EPA significantly inversely associated with fatal prostate cancer (HR 0.82 95% CI 0.64–1.04, *p* = 0.02)	Age, race, family history of prostate cancer, education, marital status, PSA testing in the past 3 years, physical activity, smoking, diabetes, BMI, energy, alcohol, and intake of tomatoes
**Shanghai Women’s Health Study** [Murff et al. (61) and Murff et al. (62)]	Breast	71,859	712	8	Significant interaction between *n*-6 PUFA and marine-derived *n*-3 (*p* = 0.01). Women with lower intake of long chain *n*-3 PUFAs and higher intake of *n*-6 PUFA had increased risk for breast cancer vs. women with higher intake long chain *n*-3 PUFAs and lower intake of *n*-6 PUFAs (RR 2.06; 95% CI = 1.27–3.34)	Age, age at menopause, alcohol, BMI, smoking, family history of breast cancer, diabetes, total red meat consumption, total fish consumption, total vitamin E, age at first pregnancy, parity, physical activity, education, and HRT
	Colorectal	73,243	396	NA	AA significantly positively associated with risk (RR 1.39 95% CI 0.97–1.99, *p* = 0.03). Non-significant positive association between higher *n*-6:*n*-3 ratio and risk (RR 1.95 95% CI 0.97–1.99, *p* = 0.19)	Age, BMI, energy-adjusted *n*-3 (g/day), energy-adjusted *n*-6:*n*-3 ratio, smoking, alcohol, physical activity, energy-adjusted red meat intake (g/day), menopausal status, HRT, multivitamin use, energy, and aspirin use
**Multiethnic Cohort Study** [Park et al. (57)]	Breast	85,089	3,885	12.4	No significant association and no ethnic-specific association associations observed	Age, ethnicity, alcohol, BMI, smoking, family history of breast cancer, age at menarche, age at first child birth, number of children, age at and type of menopause, HRT, energy, and education
**E3N** [Thiébaut et al. (56)]	Breast	56,007	1,650	8	Significant interaction between *n*-6 and marine-derived *n*-3 PUFA (*p* = 0.042). Women with higher *n*-6 had reduced risk if they had marine-derived *n*-3 PUFAs (HR 0.62 95% CI 0.44–0.86, *p* = 0.021)	Age, menopausal status, alcohol, BMI, smoking, family history of breast cancer, personal history of benign breast disease, age at menarche, parity, age at first full-term delivery, and HRT
**CPS-II** [Daniel et al. (58)]	Colorectal	43,108 men and 55,972 women	348 men and 337 women	NA	In women, non-significant positive association between total *n*-3 (driven by ALA intake) and risk of colorectal cancer (RR 1.38 95% CI (1.02–1.85, *p* = 0.09). In men, non-significant inverse associations with total *n*-6, total *n*-3, and ALA for and risk (RR 0.81 95% CI 0.61–1.07, *p* = 0.07; RR 0.86 95% CI 0.66–1.13, *p* = 0.09; RR 0.87 95% CI 0.87, 0.66–1.114, *p* = 0.09, respectively)	Age, HRT (in women only) recreational physical activity, NSAID use, colorectal screening, BMI, energy, red and processed meat, low-fat dairy, fruit, and vegetable intake
**VITAL** Sczaniecka et al. (59)	Breast	30,252	772	6	EPA and DHA were significantly inversely associated with risk for breast cancer (HR 0.70 95% CI 0.54–0.90, *p* = 0.04; HR 0.67 95% CI 0.52–0.87)	Age, race, age at menopause, alcohol, height, BMI, family history of breast cancer, age at menarche, hysterectomy, HRT, history of mammogram screening, history of benign breast biopsy, NSAIDS, physical activity, energy intake, fruit and vegetable intake and education
**Japan Public Health Center (JPHC)–Based Prospective Study** [Sasazuki et al. (63)]	Colorectal	41,382 men and 47,192 women	521 men and 350 women	9.3	In men, EPA, DPA, marine-derived *n*-3 PUFA, total *n*-3, and total *n*-6 significantly inversely associated with invasive proximal colon cancer risk (RR 0.27 95% CI 0.11–0.66, *p* = 0.01; RR 0.35 95% CI 0.14–0.88; RR 0.42 95% CI 0.18–0.98; HR 0.42 95% CI 0.18–0.98; RR 0.46 95% CI 0.21–0.99, *p* = 0.04, respectively). Higher *n*-3:*n*-6 ratio significantly positively associated with rectal cancer (RR 1.62 95% CI 0.89–2.93). In women, an overall significant inverse association between EPA, DHA, DPA, marine-derived *n*-3 PUFA, and risk for colorectal cancer (RR 0.49 95% CI 0.27–0.89, *p* = 0.01; RR 0.50 95% CI 0.28–0.90, *p* = 0.01; RR 0.53 95% CI 0.29–1.00, *p* = 0.04; RR 0.60 95% CI 0.31–1.14, *p* = 0.04) EPA non-significant inverse association with proximal colon cancer (RR 0.45 95% CI 0.20–1.05, *p* = 0.07). DPA significantly inversely associated with proximal colon cancer (RR 0.37 95% CI 0.16–0.85, *p* = 0.02)	Age, BMI, smoking, alcohol intake, current or past use of medication for diabetes, physical activity, colorectal screening, energy, energy-adjusted intake of calcium, vitamin D, fiber, and red meat

The four studies on breast cancer produced inconsistent findings. In the Multiethnic Cohort Study, no association between PUFAs and risk for breast cancer was observed (57). However, the E3N and the Shanghai Women’s Health Study cohorts, both observed statistically significant interactions between *n*-6 PUFAs, total marine-derived *n*-3 PUFAs, and risk for breast cancer (60, 61). Findings from these studies suggest that women with the lowest intake of total marine-derived *n*-3 PUFAs and the highest intake of *n*-6 PUFAs had increased risk for breast cancer compared to women with the highest intakes of total marine-derived *n*-3 PUFAs and the lowest intake of *n*-6 PUFAs (60, 61). The only investigation in breast cancer to consider both dietary PUFAs and fish oil supplementation, a commonly consumed supplement in the US (64), was conducted by Sczaniecka et al. in the VITAL study (59). Initial analyses based on dietary intake showed a trend toward a protective association for both EPA and DHA and breast cancer risk. Additional analyses, that included intake of EPA and DHA from diet and supplementation with fish oil, showed that both EPA and DHA were significantly associated with reduced risk for breast cancer (59).

Results from the cohort studies on colorectal cancer also are conflicting. Since gender and cancer site (colon or rectum) have influenced findings of previous studies, we also have taken these factors into account in our review of the literature (65). In the Shanghai Women’s Health Study, a significant positive trend was observed for AA and risk for colorectal cancer across quintiles (62). When stratified by cancer site (colon or rectal), a non-significant positive trend was observed for the ratio of *n*-6:*n*-3 and risk for colon cancer (62). In the JPHC study, a population characterized by high fish intake, there was no association between PUFAs and overall risk for colorectal cancer in men. However, significantly protective associations were found between EPA, DPA, total marine-derived long chain PUFAs, and total *n*-3 and invasive cancer specific to the proximal colon (63). Surprisingly, a significant protective effect was observed for higher total *n*-6 and invasive cancer of the proximal colon, whereas as increased risk for rectal cancer was observed for a higher *n*-3:*n*-6 ratio. In women, EPA, DHA, DPA, and total long chain PUFAs were associated with a significant reduction in risk for colorectal cancer. In further analysis stratified by cancer site, there was a non-significant trend for EPA to be protective and a significant protective effect seen for DPA, respectively, for invasive cancer of the proximal colon. Study findings from the CPS-II showed no overall significant associations between PUFAs and risk for colorectal cancer in men or women. However, a non-significant trend was observed for increased risk with higher total *n*-3 PUFAs in men and non-significant protective associations were seen for women with higher intakes of ALA and total *n*-3. Surprisingly, total-*n*6 was also associated with a non-significant protective effect (58).

Single studies in pancreatic and prostatic cancer were conducted using data from the NIH-AARP cohort. For pancreatic cancer, higher intakes of AA, DHA, and total *n*-3, driven by ALA intake, were significantly associated with risk for pancreatic cancer in men and women combined (56). For prostate cancer, there was no association between PUFAs and risk for disease; however, a significant positive association was found between ALA and risk for aggressive prostate cancer. Higher intakes of EPA were significantly associated with lower prostate cancer mortality (55).

### Clinical trials which contribute to our understanding of the potential use of PUFAs in cancer prevention and control

As noted previously, because PUFAs have pleotropic effects at the cellular and molecular level, their efficacy has been tested in clinical studies. Supplementation with *n*-3 PUFAs has been investigated in clinical trials to assess whether PUFAs can directly benefit patients with cancer. We identified nine clinical trials that were published from 2008 to present that investigated effects of *n*-3 PUFAs on cancer patient populations. The details of these studies and their findings are described in Table 2. Briefly, three investigations were conducted in the US (66–68), three in Europe (69–71), and a single study in Canada (72) as well as Brazil (73). All studies involved an intervention group who received either *n*-3 supplements (pills, capsules, or supplemented oral feedings) containing fish oil (66, 68–73) or food sources high in *n*-3 PUFAs (67). The amount and type of *n*-3 PUFAs varied between studies and all studies included a control arm. All studies determined physiological levels of PUFAs in blood or relevant tissues to assess adherence with the study regimen and bioavailability of the *n*-3 PUFAs. Various outcome variables were studied including biomarkers of inflammation (69–71, 73), tumor proliferation (66, 67), gene expression (68), and cancer prognosis (72).

**Table 2:** **Clinical trials reporting the effects of PUFAs on cancer patients**.

Organ	Population	Sample size	Approximate length of study	Intervention	Relevant findings
**Lung**	Patients with NSCLC undergoing chemotherapy [Murphy et al. (72) and Murphy et al. (75)]	46	1 year	Intervention: fish oil supplement, choice of liquid or capsules, 2.20 g EPA, 0.24–0.50 g DHA; control: SOC	Plasma EPA and DHA higher in intervention (*p* < 0.001 and *p* = 0.04); intervention had higher response rate to chemotherapy (*p* = 0.01), greater clinical benefits (*p* = 0.02), and greater 1 year survival than SOC group (*p* = 0.15)
	Patients with IIIa N2-IIIb NSCLC undergoing chemotherapy [van der Meij et al. (74) and van der Meij et al. (70)]	40	5 weeks	Intervention: protein and energy dense oral nutritional supplement, ∼1.01 g EPA, ∼0.46 g DHA; control – isocaloric oral nutritional supplement, 400 mL ensure	Plasma EPA and DHA higher in intervention (*p* = 0.06); no differences between groups in [AA] (*p* = 0.65); intervention had lower IL-6 production in *ex vivo* stimulation vs. control (*p* = 0.08); no difference between groups for serum IL-6, CRP, sTNF-p55, albumin, and HLA-DR expression on monocytes; EPA and DHA in intervention inversely correlated with IL-6 and CRP (Pearson *r* = −0.80, *p* = < 0.05); intervention vs. control higher scores on: global health status (*p* = 0.04), physical function (*p* < 0.01), cognitive function (*p* < 0.01), and social function (*p* = 0.04)
	Patients with advanced inoperable NSCLC undergoing chemotherapy [Finocchiaro et al. (69)	33	10 weeks	Intervention: *n*-3 supplement, capsules, 0.51 g EPA, 0.34 g DHA; control: placebo, 0.85 g olive oil	EPA higher in plasma and erythrocytes, DHA higher in plasma only for intervention (*p* < 0.05 for all); IL-6 and CRP levels lower in intervention (*p* < 0.05); IL-6, CRP, and PGE2 decreased from baseline in intervention (*p* < 0.05); hydroxynonenal and ROS lower for intervention (*p* < 0.05)
**Prostate**	Patients localized prostate cancer awaiting prostatectomy [Aronson et al. (66)]	48	4–6 weeks	Intervention: 15% total energy from fat + fish oil supplement, 1.00 g EPA, 1.80 g DHA, *n*-6: *n*-3 ratio 2:1; control: 40% total energy from fat, *n*-6: *n*-3 ratio 15:1	Lower *n*-6:*n*-3 ratio in intervention for benign and malignant prostate tissue and RBCs (*p* = 0.042, *p* < 0.001, and *p* < 0.001, respectively); reduced tumor proliferation rate (Ki67) in intervention (*p* = 0.026); no difference between groups for serum IGF-1, IGFBP-1, IGFBP-3, PSA, urine PGEM, or prostatic prostaglandin E2, COX-2, angiogenesis (CD31), or apoptosis (TUNEL)
	Patients localized prostate cancer awaiting prostatectomy [Demark-Wahnefried et al. (67)]	161	31 days	Intervention: flaxseed, 30 g; low-fat diet,<20% total energy from fat; flaxseed + low-fat diet; control: usual diet	Interventions with flaxseed had higher EPA in erythrocytes and prostate tissue and decreased *n*-6:*n*-3 ratio in prostate tissue (*p* < 0.05); reduced tumor proliferation rate (Ki67) in interventions with flaxseed (*p* < 0.05); no difference between groups for apoptosis (TUNEL), serum SHBG, free androgen index, IGF-1, IGFBP-3, and CRP
	Patients with low burden prostate cancer following active surveillance treatment protocol [Magbanua et al. (68) and Chan et al. (76)]	84	12 weeks	Interventions: lycopene supplement, 30.0 mg + fish oil placebo (olive oil); fish oil supplement, 1.10 g EPA, 0.54 g DHA + lycopene (soy oil); control: soy or olive oil) *standard multivitamins consumed by all	No difference in change in IGF-1 or IGF-1R gene expression between placebo and lycopene intervention; no change in COX-2 gene expression for placebo and fish oil intervention; greater increase in IGF-1 for those with initially high tomato intake (*p* = 0.01); Androgen and estrogen metabolism modulated for men with initially high tomato and fish intake (*p* < 0.05); modulation of DHA and insulin receptor signaling for men with initially high fish intake (*p* < 0.05); modulated AA metabolism in fish oil intervention compared to control (*p* = 0.01); modulation of Nrf2-mediated oxidative stress response pathway for fish oil and lycopene interventions (*p* < 0.01)
**G.I**.	Patients undergoing surgery [Sultan et al. (71)]	195	2 weeks (7 days pre- and post- surgery)	Intervention: liquid feeding enriched with *n*-3 PUFAs, 3.00–6.00 g EPA, 1.30–2.60 g of DHA; controls: standard enteral nutrition, pre and postsurgery; isotonic liquid feed, post-surgery only	Plasma and lymphocyte EPA and DHA higher in intervention (*p* < 0.01); plasma *n*-6:*n*-3 ratio, LA, and AA lower in intervention (*p* < 0.001, *p* = 0.019, and *p* = 0.018); increase in HLA-DR expression on monocytes for intervention group pre surgery (*p* < 0.001); no significant differences between groups for HLA-DR expression on monocytes or T-lymphocytes or in CRP
	Patients receiving chemotherapy after surgery [Bonatto et al. (73)]	38	8 weeks	Intervention: fish oil supplement, 0.30 g EPA, 0.40 g DHA; control: no supplement	EPA and DHA increased in blood polymorphonuclear cells (PMNC) in intervention vs. control (*p* < 0.05); ratio of AA: EPA decreased in intervention and lower than control (*p* < 0.05); increased number of PMNCs in intervention (*p* < 0.05); increased PMNC function (*p* < 0.05)

Indeed, studies of PUFA supplementation show that physiologic levels of PUFA are highly responsive to changes in diet, and dosing studies have been performed in healthy subjects, as well as in patients with lung cancer undergoing chemotherapy (69, 70, 72). Supplementation also is accompanied by changes in inflammatory markers. Van der Meji et al. reported decreased production of IL-6 from white blood cells and significantly lower plasma IL-6 and C-reactive protein (CRP) levels in patients supplemented with EPA compared to baseline (70). This same investigation also reported significant improvements in physical and cognitive functioning in the intervention arm as compared to controls (74). Similarly, in a patient population with reduced life expectancy, Murphy and colleagues observed increased plasma levels of EPA and DHA in patients who were supplemented compared to controls and these levels were associated with completion of more cycles of chemotherapy and better response to treatment and improved 1-year survival (72). The investigators also observed that patients in the intervention group maintained their body weight and patients with the greatest increases in plasma EPA gained muscle mass, in contrast to the control group which had significant weight loss and muscle loss (75). Finocchiaro et al. also observed significant increases in plasma EPA and DHA with supplementation, but erythrocyte membrane composition showed increases only in EPA. Over the course of the study, CRP, IL-6, and TNF-alpha concentrations increased in the controls and PGE2 did not change, whereas there were significant decreases in these biomarkers in the intervention arm with the exception of TNF-alpha which did not change (69). Similarly, in a group of patients undergoing chemotherapy for gastrointestinal cancer, Bonatto and colleagues reported that following fish oil supplementation there was increased EPA and DHA and decreased AA in peripheral mononuclear cells (PMNC) which correlated with an increased number of PMNCs, improvements in immune function, and significant increases in body weight (73). Conversely, patients undergoing subtotal esophagectomy or gastrectomy who received pre- and post-operative supplementation with EPA and DHA had similar immunological response and clinical course compared to controls, despite having increased plasma and lymphocyte composition of EPA and DHA (71).

As noted earlier, PUFAs can mediate cell proliferation and apoptosis in cancer cells. Three recent randomized clinical trials (RCT) tested the effects of PUFAs on men with localized prostate cancer. In a group of 84 men on active surveillance, Magbanua et al. showed that fish oil supplementation for 3 months did not have an effect on global gene expression in normal prostate tissue (68). Yet, exploratory pathway analyses suggested that fish oil modulated Nrf2-mediated oxidative stress and metabolism of AA. Additional analysis showed that fish oil supplementation did not affect COX-2 gene expression suggesting that perhaps the alteration in AA metabolism may have been modulated through the 5-LOX pathway although this was not investigated (76). In a phase II RCT among men awaiting prostatectomy, supplementation with fish oil for ∼28 days resulted in higher levels of EPA and DHA and reduced levels of *n*-6 PUFAs in erythrocytes and surgically excised prostatic tissue (66). While no differences in PGE_2_, COX-2, angiogenesis, or apoptosis were observed; tumor cell proliferation (Ki67) was significantly lower in the intervention arm compared to controls (66). Using a similar study design, Demark-Wahnefried et al. found that following supplementation with 30 g/day of flaxseed (a rich source of ALA) for ∼30 days prior to prostatectomy; there were significantly higher levels of EPA in erythrocytes and prostatic tissue in the intervention arm. No difference in apoptosis was observed between the flaxseed arm and controls; however, tumor proliferation rates were significantly lower in the intervention arm. Additional analyses investigated the associations between prostatic PUFAs, and genetic variation in *FADS* genes with tumor proliferation and serum prostate specific antigen (PSA) in order to elucidate the anti-proliferative effects of flaxseed (77). Surprisingly, a positive correlation between ALA and tumor proliferation and serum PSA and no association between other PUFAs and these markers was observed. These associations appeared to be independent of the amount of ALA consumed, suggesting that the metabolism of ALA was altered in the target tissue. This was confirmed in additional analyses which also showed significant interactions between SNPs in the *FADS2* gene, ALA and tumor proliferation, and serum PSA (77). Based on these findings, future studies are needed to better understand whether altered PUFA metabolism is a driver or passenger for prostate cancer. If altered PUFA metabolism is found to drive cancer progression, this could provide opportunities to investigate the efficacy of personalized-diets with modified dietary PUFAs as a complementary therapeutic option.

## Conclusion and Future Directions

In summary, the results from pre-clinical studies provide compelling evidence that PUFAs can mediate cancer progression *in vitro* and *in vivo* in models of several different types of cancer. Mediation may occur through several mechanisms including regulation of gene expression, angiogenesis, cell migration, and apoptosis. Additionally, DHA may have chemo-sensitizing effects in humans, but this needs to be confirmed in larger studies. Based on the current literature, the pro-inflammatory and anti-inflammatory characteristics of PUFAs are the key underlying mechanisms mediating their biological effects. While these findings have expanded our knowledge of the molecular targets of PUFAs, the extent to which they are generalizable to humans remains unclear. Studies that employ well-characterized cell lines and animal models provide insight into how PUFAs affect cells with specific and known mutations; however, cancer is a heterogeneous disease and thus human tumors may be very different from one person to another. Genetic/genomic differences in tumors can produce differences in tumor phenotype resulting in differences in PUFA metabolism. For example, mutations in *FADS2* has been reported in tumors from breast cancer patients and the loss of expression of *FADS2* was significantly associated with aggressive tumor phenotype and reduced survival (78). The *FADS* cluster localizes to a genomic region that has been associated with breast, colon, and prostate cancer (79). Understanding these genetic and genomic differences and how they affect nutrient metabolism could allow us to begin to experiment with altering dietary intakes of PUFAs to test the effects on cancer progression.

Taken as a whole, the findings from cohort studies suggest that dietary PUFAs play a role in cancer risk and progression; however, no clear pattern has emerged. This may be due to the well documented difficulties in assessing dietary intake that include poor participant recall and elevated measurement error (80) but may also involve other factors. As noted in Table 1, only one study considered additional intake of PUFAs from fish oil supplements and this study found that EPA and DHA were significantly associated with reduced risk for breast cancer (59). This highlights the importance of considering supplementation and also the hypothesis that large amounts of *n*-3 PUFAs, typically not seen in US populations, are needed to affect cancer risk. In support of this hypothesis, the study conducted in a Japanese population with high fish intake also found significant protective effects for marine-derived PUFAs. Another factor associated with discordance between dietary intake of PUFAs and cancer is genetic variation. As noted previously, SNPs in the genes involved in PUFA metabolism play a critical role in determining PUFA metabolism explaining up to 28 and 12% of the variance in plasma levels of AA and LA, respectively (9). Thus, genetic variation could be a major factor potentially explaining the discordance between dietary intake and cancer in population studies.

Collectively, the reports from recent clinical trials support many of the findings from pre-clinical studies. Overall, cancer patients with different types of malignancies and undergoing different treatments appeared to benefit from PUFA supplementation. Of note is the relative short time frame that patients consumed PUFAs yet achieved benefits in immunological or inflammatory markers and physical and psychological measures as well as prognosis. However, it should be noted that clinical trials provided large amounts of *n*-3 PUFAs, far greater than what is typically consumed in the diet. Thus, larger intakes of *n*-3 PUFAs may be required in order to receive the full anti-carcinogenic benefits of these compounds. Particularly noteworthy are the results from studies in prostate cancer which indicate that fish oil supplementation modulates AA metabolism in prostatic tissue and inhibits tumor proliferation in men with localized disease. Although no direct correlation between PUFA levels in the target tissue and outcomes were made in these studies, fish oil appears to provide some direct protective effects on prostatic tumors. Further, findings that ALA and SNPs in the genes involved in PUFA metabolism were positively associated with markers for aggressive disease showed that genetic variation should be considered further as it plays an important role in determining PUFA metabolism (9, 81, 82). It is possible that gene-nutrient interactions in the PUFA pathway likely portend risk for cancer and aggressive disease; however, further studies are needed in this area (83).

In conclusion, PUFAs are biologically active food components that are consumed daily from a variety of food sources. Individual PUFAs produce prostaglandins and leukotrienes with distinct biological functions that elicit pro- and anti-inflammatory responses through several signaling pathways that regulate cell proliferation, apoptosis, and angiogenesis. The metabolism of PUFAs is complex and controlled by enzymes that are highly polymorphic and map to a genomic region frequently associated with cancer. Thus, to better delineate the associations between PUFAs and cancer in humans, future studies should consider dietary intake of PUFAs, and variation in genes encoding the enzymes in PUFA metabolism and the potential for gene-nutrient associations between SNPs and PUFAs. In addition, because the expression of genes encoding the enzymes in the PUFA pathway is frequently lost in the target-tissue, metabolism of PUFAs in tumor tissues may be altered and this needs to be considered. The findings from such studies could allow for the identification of individuals with altered PUFA metabolism that may benefit from personalized diets.

## Conflict of Interest Statement

The authors declare that the research was conducted in the absence of any commercial or financial relationships that could be construed as a potential conflict of interest.
